# South Africa’s new standard material transfer agreement: proposals for improvement and pointers for implementation

**DOI:** 10.1186/s12910-020-00526-x

**Published:** 2020-09-03

**Authors:** Donrich W. Thaldar, Marietjie Botes, Annelize Nienaber

**Affiliations:** 1 School of Law, University of KwaZulu-Natal, Durban, South Africa; 2 African Health Research Flagship, University of KwaZulu-Natal, Durban, South Africa; 3 Faculty of Law, University of Pretoria, Pretoria, South Africa; 4 Centre for Ethics and Philosophy of Health Sciences, Faculty of Health Sciences, University of Pretoria, Pretoria, South Africa; 5 Division of Law, Abertay University, Dundee, Scotland, UK

**Keywords:** Human biological material, Material transfer agreement, MTA, Research ethics, Intellectual property rights, Benefit-sharing, Consent, Privacy

## Abstract

**Background:**

Whenever South African (SA) research institutions share human biological material and associated data for health research or clinical trials they are legally compelled to have a material transfer agreement (MTA) in place that uses as framework the standard MTA newly gazetted by the South African Minister of Health (SA MTA).

**Main body:**

The article offers a legal analysis of the SA MTA and focuses on its substantive fit with the broader legal environment in South Africa, and the clarity and practicality of its terms. The following problematic aspects of the SA MTA are highlighted: (a) Where only data and no human biological material are transferred, the SA MTA does not apply, leaving a lacuna; (b) Health Research Ethics Committees are required to be parties to a MTA despite it being outside their legal mandate and undermining their oversight function; (c) the SA MTA’s consent provisions are not aligned with extant law; and, similarly, (d) its provision on donor ownership is misaligned with extant law; (e) its creation of fictitious performance can only cause frustration on the part of an injured party; (f) its benefit-sharing provision is vague and will have little practical effect; (g) its dispute-resolution provisions fail to adequately protect South African research institutions and research participants; (h) it fails to provide substantive guidance regarding intellectual property as its provisions relating to intellectual property may cause practical problems; and, finally, (i) its data privacy provision is insufficiently specific, is overbroad, and fails to provide terms that in general would facilitate the international sharing of human biological material and associated data in terms of existing privacy law.

**Conclusions:**

While some of the problematic aspects of the SA MTA are intricate and require consultative processes with stakeholders and others, to develop comprehensive solutions, most of the problematic aspects can be resolved immediately through amendments by the South African Minister of Health. The formulation of such amendments is proposed and, where possible, interim measures are suggested that may ameliorate the problems presented by the SA MTA.

## Background

Because biomedical researchers rely heavily on biological and bio-informatic resources that are collected and created by others, there is growing need to share these resources [1]. A resource shared frequently and almost routinely is that of human biological material and its associated data. However, this commonplace sharing of human biological material and associated data raises a myriad of legal and ethical issues in terms of this shared relationship. For example, because research potentially can lead to valuable discoveries, the ownership and control of downstream discoveries can become contentious. In these circumstances, how are the legal rights and duties between the sharing researchers to be determined?

### Material transfer agreements

A material transfer agreement (MTA) is a written agreement between two research institutions, one being the provider (Provider) of human biological material and the other the recipient (Recipient) that intends to use this material for research purposes. A MTA governs the transfer of tangible research materials such as reagents, cell lines, plasmids, vectors and any progeny, derivatives or modifications between the parties. The transfer of associated data may be regulated in a MTA or in a separate data transfer agreement (DTA). MTAs range from short, simple agreements to complex agreements that involve lengthy and costly negotiations [2].

Primarily to save on the transactional cost involved in negotiating custom MTAs, there have been various attempts to develop standardised MTAs for international use. Perhaps the most well-known example is the Uniform Biological Material Transfer Agreement (UB MTA) that was developed by the National Institutes of Health (NIH) in collaboration with research institutions. The UB MTA has been in use for over a generation and has been adopted by 684 institutions from all over the world [3]. An institution that adopts the UB MTA signs a master agreement which is filed at a central repository. Institutions that are UB MTA signatories in transferring material between one another need merely to execute a standard Implementing Letter for each transfer [4]. The UB MTA was designed for the transfer of proprietary biological materials [5], but it is worth noting that the NIH also developed a stand-alone MTA template specifically for use in transferring human biological material [6]. Further, a number of other prominent organisations have standardised MTAs. An example is the World Health Organisation (WHO) that uses Standard Material Transfer Agreement 2 (SMTA 2) for the sharing of human biological materials with institutions as part of their Pandemic Influenza Preparedness Framework [7].

There is a growing international concern that research specimens and data are not shared optimally globally [8]. The need to provide greater access to research data sets has been articulated in a number of documents, including the OECD Principles and Guidelines for Access to Research Data from Public Funding [9], the Toronto Statement [10], and the Global Alliance for Genomics and Health’s White Paper [11]. A major obstacle to more effective sharing across national borders is contradictory legal and ethical frameworks [8]. What is required legally and is viewed as ethical in one country may be regarded completely differently in another country. To facilitate the movement to provide greater global access in sharing bio-resources, a group of bioethicists have initiated a project to develop an international Charter of Principles for Sharing Data and Bio-Specimens [8] which aims to overcome the obstacle of contradictory domestic legal and ethical frameworks, and which includes a template MTA and DTA [8].

### Legal development in South Africa

The high genetic diversity of Southern African populations is well-known. Consequently, the large-scale collection of human biological material from South Africa (and the data subsequently derived) hold a promise of making a huge impact on the prevention, diagnosis and treatment of disease – not only in South Africa but worldwide. Unsurprisingly, the South African research community is part of numerous international collaborative research projects that involve the sharing of human biological material and associated data, such as H3Africa, B3Africa, HapMap, and MalariaGEN [12].

Against this background, in July 2018 the South African Minister of Health (the Minister) published a standard MTA (SA MTA) in the *Government Gazette* and gave notice that South African research institutions sharing human biological material for health research or clinical trials must have a MTA in place based on the SA MTA [13]. This development makes South Africa unique in the world as the only country to require the use of a standard MTA through national legislation and forces even parties *outside* South Africa to use the SA MTA when engaging in the transfer of human biological material to or from South Africa.

In this article we offer a legal analysis of the SA MTA and concentrate on its substantive fit with the broader legal environment in South Africa, as well as on the clarity and legal practicality of its terms. Although we recognise that the topic of the SA MTA intersects with various ethical discourses such as whether research participants have a moral entitlement to benefit-sharing and the movement (referred to above) to increase global access to bio-resources, our analysis is limited to the core legal issues mentioned above.

In the following paragraphs we familiarise the reader with the legal environment related to the use of human biological material for research in South Africa and with the *ratio legis* of the SA MTA. After providing this background information, we proceed to the analysis of the SA MTA in the main body of the article.

### Legal environment in South Africa

The SA MTA was introduced into an extensive and well-developed domestic legal environment relating to the use of human biological material for research that is primarily governed by the National Health Act (NHA) [14] and its various regulations. In this section we provide the reader with a brief overview of the most salient aspects of the legal environment that are relevant to our analysis of the SA MTA below.

First, informed consent must be obtained in writing from research participants for their participation in the research project ([14] section 71) and for the removal of human biological material ([15] regulation 3(1)(a)). In addition, a researcher who conducts health-related research involving human participants (which includes research on human biological material and associated data given that these are provided by human research participants) is legally compelled, inter alia, to consult with representatives from the participating community or other relevant research stakeholders where appropriate and to disseminate the research results, negative or positive, to research stakeholders in a timely and competent manner ([16] regulation 5).

There is no legal provision per se for benefit sharing in the context of the provision of human biological material. In fact, compensation for providing human biological material for research is limited to reasonable costs incurred and ‘to receive any form of financial or other reward’ is unlawful and a criminal offence ([14] section 60(4),(5)). Also, it is important to note that persons who provide their human biological material for use in a research project legally are deemed to transfer *exclusive rights* in the human biological material to the research organisation that receives the material for use in research ([17] regulation 26). With regard to ownership of the results of research if the research received public funding, the recipient of such funding – typically a university – has a legal right to register and own the intellectual property emanating from the research ([18] section 4(1)). The recipient of public funding also has various legal duties to protect such intellectual property – even if it elects not to retain ownership in its intellectual property or not to obtain statutory protection for the intellectual property ([18] sections 4(2) and 5(1)). The possibility exists to co-own the intellectual property with a private entity if a number of criteria are fulfilled, including a contribution of resources and the co-creation of the relevant intellectual property by the private entity ([18] section 15(2)). Although research participants contribute resources (in the form of their human biological material), they do not conduct the actual research and therefore are excluded from being co-owners of intellectual property emanating from publicly-funded research using their human biological material.

Proposed health-related research studies in South Africa must be approved by a health research ethics committee (HREC) that is registered with the National Health Research Ethics Council ([14] section 73) – a statutory body appointed by the Minister of Health ([14] section 72(2)). An institution that conducts health research must either to have its own HREC or to have access to a HREC ([14] section 73(1)). Currently, there are 46 HRECs registered with the Council [19], of which about two-thirds are university-based research ethics committees; about a third are research ethics committees belonging to various other bodies involved in health research and one HREC is a private company [19].

The mandate of HRECs in terms of the NHA is to ensure that a proposed health-research study has a health-related purpose and that they meet the particular HREC’s ethical standards ([14] section 73(2)). However, the NHA further provides that the National Health Research Ethics Council must set national norms and standards ([14] section 72(6)(c)). The most pertinent source establishing such standards that was produced by this Council and subsequently was issued by the Department of Health is a document titled *Ethics in Health Research: Principles, Processes and Structures* (Department of Health Ethics Guidelines) [20]. These Guidelines provide that the primary role of HRECs is to protect the interests of research participants ([20] p40).

It is important to note that the Department of Health Ethics Guidelines are not only binding on HRECs, but effectively have the force of law and apply to all research on human biological material and associated data. This is because the Regulations relating to Research with Human Participants provide that all research with human participants (which includes research on human biological material and associated data, given that these are provided by human research participants) must comply with ethics guidelines issued by the Department of Health ([16] regulations 2(a), 6(b)).

An important issue dealt with in the Department of Health Ethics Guidelines is the nature of informed consent. The Guidelines provide that the following forms of consent are acceptable ([20] p31):

- ‘Narrow (restrictive) consent’, which is described as ‘the donor permits use of the biological specimen for single use only; no storage of leftover specimen; and no sharing of data or specimen. This form necessitates new consent if further use is desirable’. Consent to a ‘single use’ – a *specific* research purpose – is more commonly referred to as ‘specific consent’.
- Tiered consent, which is described as ‘the donor provides consent for the primary study and chooses whether to permit storage for future use, sample and data sharing’.
- Broad consent, which is described as ‘the donor permits use of the specimen for current research, for storage and possible future research purposes, even though the precise nature of future research may be unclear at present’.

Unrestricted or ‘blanket’ consent is ‘not recommended’ ([20] p31). There is also a clear *preference* for broad consent: The Guidelines state that consent should be ‘broad enough to allow for future and secondary uses of data, in line with the opportunities to use such data in advancing knowledge to improve health’ ([20] p31).

These provisions on consent contained in the Department of Health Ethics Guidelines may be disrupted by the Protection of Personal Information Act (POPIA) [21]. Although POPIA was enacted in 2013, provisions relevant to research participant consent only have entered into force on 1 July 2020 [22]. Furthermore, given that POPIA contains a one-year grace period ([21] section 114(1)), its actual enforcement date will only be from 1 July 2021. In the following paragraphs, we provide a brief overview of the relevant provisions of POPIA.

POPIA will govern the processing of personal information. ‘Processing’ is defined as including, inter alia, the collection, storage, use, dissemination, distribution, and destruction of information ([21] section 1); ‘personal information’ is defined as including, inter alia, physical or mental health information, and biometric information, which, in turn, includes DNA analysis ([21] section 1). Accordingly, the data associated with human biological material in the context of health research would in all likelihood fall within the ambit of POPIA. Also, to the extent that human biological material will be used for genetics and genomics research, human biological material *itself* can be perceived as a container of biometric information, and would therefore also fall within the ambit of POPIA [23].

POPIA provides that personal information must be processed according to eight conditions for processing ([21] section 4(1)). Most pertinent for present purposes are the following conditions:

- The ‘processing limitation’ condition provides that personal information may only be processed if a legal ground for processing is present ([21] sections 9–12). One of these legal grounds – and probably the only one applicable in the context of health research – is consent by the ‘data subject’ (the research participant in the health research context). Consent is defined as ‘any voluntary, *specific* and informed expression of will in terms of which permission is given for the processing of personal information’ ([21] section 1, emphasis added).
- The ‘purpose specification’ condition provides that personal data ‘must be collected for a *specific*, explicitly defined and lawful purpose’ ([21] section 13, emphasis added).
- The ‘further processing limitation’ condition provides that there can be ‘further processing’ of personal information under certain circumstances, including, inter alia, when such ‘further processing’ is for purposes of *research*, subject to the condition that the further processing is carried out solely for the research purpose and the personal information may not be published in any identifiable form ([21] section 15).

Worth noting is that a person’s health and biometric information also qualifies as ‘special personal information’ in terms of POPIA ([21] section 26(a)). To process special personal information, a legal ground for processing must be present from an additional set of possible legal grounds ([21] section 27(1)). There is some overlap between the legal grounds for processing personal information, and for processing special personal information, such as *consent* by the ‘data subject’. Again, the meaning of consent is determined by its definition, namely ‘any voluntary, *specific* and informed expression of will in terms of which permission is given for the processing of personal information’ ([21] section 1, emphasis added). Another legal ground for processing special personal information that is pertinent in the present context is research, provided that (a) such research serves a public interest, or that (b) it is impossible or would involve a disproportionate effort to ask for consent to such research, and sufficient guarantees are provided for to ensure that the processing does not adversely affect the research participant’s privacy to a disproportionate extent ([21] section 27(1)(d)).

Despite the clear and unambiguous definition of consent as a *specific* expression of will, some have argued that POPIA may be interpreted as allowing *broad* consent, based on the legal doctrine of purposive interpretation [24]. We have reservations about the legal merits of such an argument. The South Africa’s Constitutional Court has held that a ‘purposive reading of a statute must of course remain faithful to the actual wording of the statute’ [25]. Accordingly, an attempt to change the meaning of the word ‘specific’ as used in POPIA to ‘broad’ seems to be based on an over-enthusiastic reliance on purposive interpretation [26]. This debate about interpretation aside, POPIA itself makes provision that the Information Regulator (POPIA’s enforcement mechanism) can exempt persons from having to comply with the eight conditions for processing personal information ([21] section 37(1)(a)); also, POPIA provides that the Information Regulator may authorise a person to process special personal information, subject to any conditions that may be specified ([21] section 27(2)). We suggest that a case *potentially* can be made (in terms of sections 37(2)(e), (f) and 27(2)) for such an exemption and authorisation for persons engaging in health research. Accordingly, the actual impact of POPIA on the kind of consent required in South Africa from health research participants will only become clear once the Information Regulator has considered and decided whether to grant an exemption and an authorisation, as well as the scope and conditions of these, if granted. Given the present uncertainty we will refrain from analysing the SA MTA from the perspective of the consent provisions of POPIA.

Another important aspect of POPIA that will not be subject to a potential exemption or authorisation is its export provisions ([21] sections 72, 57). First, a Provider may only transfer personal information to a Recipient in a foreign country if a legal ground for such transfer is present ([21] section 72). These legal grounds include, inter alia, consent by the ‘data subject’, and that the law in the jurisdiction of the Recipient or the contract between the Provider and the Recipient must provide an ‘adequate’ level of protection for the processing of personal information ([21] section 72). Secondly, when a Provider in South Africa intends to transfer special personal information to a Recipient in a foreign country that does not provide an ‘adequate’ level of protection, the Provider must obtain prior authorisation for the intended transfer from the Information Regulator ([21] section 57(1)(d)). A failure to obtain prior authorisation will constitute a criminal offence ([21] section 59). However, if the Information Regulator has approved a code of conduct for the relevant sector, the need for prior authorisation is obviated ([21] section 57(3)).

The import and export of human biological material more generally are regulated by a dedicated set of regulations [27]. These regulations provide, inter alia, that a permit from the Director-General of the Department of Health is required for the import and export of human biological material ([27] regulation 2(1)). As well, these regulations lay down numerous general requirements for the import and export of human biological material, including, pertinently, that written proof must be provided that the human biological material that is to be exported was donated in terms of the NHA and will be used in terms of the NHA ([27] regulation 3(1)).

The Department of Health Ethics Guidelines provide that whenever human biological material is shared between institutions, a written agreement must be in place, but give no guidance regarding the content of such a written agreement ([20] p37). This point is a lead into the next section, namely the rationale for the SA MTA.

### Rationale for the SA MTA

The SA MTA does not have a preamble but tersely states under the heading ‘Objective’ that its objective is ‘to set out a framework within which the Parties will engage in the transfer, use and other processing of the Materials’. Therefore, it appears the SA MTA aims to provide minimum content and principles for MTAs that fall within its scope of application. Other than this statement the SA MTA provides no indication as to its substantive objectives.

As discussed above, the typical rationale for having a standard MTA is to save transactional costs and time. However, this rationale may be less prominent – if relevant at all – in the context of the SA MTA. The Minister might have had other reasons for promulgating the SA MTA as has been suggested in the academic literature. These reasons include the following: (a) There is a history of accusations of unethical or illegal conduct by health researchers regarding the use of South African – and more generally African – human biological material [28]; (b) there is a movement against so-called ‘research imperialism’ that refers to situations where researchers in the Global North undertake research in the Global South but do not share the benefits of that research in a fair way with research participants and scientific collaborators in the Global South [28]; (c) many cultures in South Africa attach particular significance to human biological material [29]; and (d) MTAs used in the international sphere are perceived not to address legal concerns specific to South Africa [29]. Clearly, these reasons point to a desire for a more protectionist national legislation, in seeming contrast with the movement (referred to above) to promote greater access to bio-resources elsewhere. These reasons would explain the decision that South Africa needed a national standard MTA and that the use of a national standard MTA should be a legal requirement. However, whether the SA MTA actually is successful in addressing the problems underlying these reasons is open to debate.

## Main text

In this section we offer a legal analysis of the SA MTA, concentrating on its substantive fit with the broader legal environment in South Africa and on the clarity and legal practicality of its terms. We highlight our concerns, propose corrective legislative measures and, where possible, propose measures that researchers may take in the interim to mitigate these concerns. Our analysis starts with the general issue of application, and then considers other issues in the order in which they arise in the SA MTA.

### Application

The notice by the Minister in the *Government Gazette* to which the SA MTA is a schedule reads as follows:

All the providers and recipients of the biological material for use in research or clinical trials under the auspices of the Health Research Ethics Committees shall use the Material Transfer Agreement of Human Biological Materials.

Accordingly, the SA MTA must be used whenever any human biological material is transferred for the purpose of research or clinical trials from the Provider to the Recipient, and – to bring the transfer under the jurisdiction of South Africa – at least one of the institutions is in South Africa. In a recent article on the SA MTA, Labuschaigne et al. suggest that the SA MTA is applicable only in the case of export, in other words when the Provider is in South Africa and the Recipient is located in a foreign jurisdiction [30]. However, the Minister’s notice does not limit the application of the SA MTA to the export of human biological material with the result all that is required to compel the use of the SA MTA is that either the Provider or the Recipient, or both, is in South Africa.

The provisions of the SA MTA are not limited to the transfer of human biological material but contain rules governing the transfer of associated data. In the SA MTA, ([13] paragraph 2.13), human biological material and associated data collectively are referred to as ‘Materials’ – the first letter is in uppercase to indicate that the word is used as defined and not in its ordinary meaning. However, the Minister’s notice in the *Government Gazette* speaks of ‘biological material’ in lowercase, indicating that these words are intended to carry their ordinary meaning. Also, it is worth noting there is no indication either in the notice or in its schedule (the SA MTA) that the definitions in the schedule are applicable to the notice, which means that the Provider and the Recipient are compelled legally to use the SA MTA only if the intended transfer contains ‘biological material’ in its ordinary meaning. In other words, if only data is transferred, the parties are not required to enter into a MTA using the SA MTA as a framework. Until POPIA fully enters into force, the sharing of only genomic data is therefore unregulated. We suggest that this issue should be rectified by the Minister in an amended notice that refers to ‘human biological material and associated data’ instead of ‘biological material’.

The SA MTA states that it sets out a ‘framework’ ([13] paragraph 1). We agree with Labuschaigne et al. that this means that the terms in the SA MTA need not be duplicated verbatim [30]. We suggest that the essence of the terms contained in the SA MTA nevertheless must be retained as a basic structure but is one on which the parties are permitted to elaborate. The practical problem, however, is that the exact extent to which parties may depart from the SA MTA is uncertain. This uncertainty presents a major problem for HRECs which, although they count having lawyers among their membership, are given the task of judging whether any particular MTA departs too far from the SA MTA. We suggest that legal certainty would be better served – and the South African research community assisted and protected – had the SA MTA indicated exactly which of its terms (or underlying principles) are compulsory and which are voluntary.

### HRECs as parties to the MTA

The SA MTA requires that a HREC registered as such with the South African National Health Research Ethics Council must be a *party* to the MTA. There are a number of uncertainties which relate to this requirement: If both the Provider and the Recipient are in South Africa, which one’s HREC should be a party to the MTA, and, if both the Provider and the Recipient are in South Africa, is it possible for both their HRECs to be parties to the MTA?

As well as these practical issues there is the more fundamental question regarding the issue of juristic personhood; being a party to an agreement requires contractual capacity and in turn requires juristic personhood. However, this requirement is problematic as the vast majority of HRECs in South Africa do not have juristic personhood. As discussed in the background, all HRECs except one are committees of larger organisations. In South African law a juristic person can be established in only three ways: (a) through specific legislation, where a statute creates a body and explicitly bestows juristic personhood on it; (b) through executive government action in terms of general legislation, for instance where the Companies and Intellectual Property Commission registers a new company; or (c) through the common law, where an organisation is established with the aim of existing as an independent legal entity that, inter alia, has the capacity to acquire its own rights and incur obligations [31]. We suggest that HRECs that are committees of research institutions do not qualify as belonging to any of these three categories: First, nothing in the NHA [14] (the statute that requires that an institution that conducts health research either has its own HREC or has access to a HREC) suggests that HRECs possess juristic personhood. Secondly, the fact that an institution that conducts health research establishes an internal committee in pursuance of legislation does not constitute action by the executive branch of government. In the case of an institution such as a university establishing a research ethics committee in pursuance of the NHA, it is no different from when a university establishes structures in pursuance of other legislation, for instance technology transfer offices (TTOs) that are established in pursuance of the requirements of the Intellectual Property Rights from Publicly Financed Research and Development Act [18]. Thirdly, HRECs that are committees of institutions are unlikely to be common law juristic persons. These HRECs function as organs of an institution rather than as independent legal entities with their own legal rights distinct from those of the institution; in other words, if a university were to stop existing, the university’s HREC also ceases. The conclusion of this analysis is that of 46 HRECs in South Africa only the one that is registered as a private company legally would be capable of being a party to a MTA. For the other 45 HRECs being a party to a MTA is a *legal impossibility*.

There is a potential counter-argument that should be considered, namely that the Minister of Health in requiring HRECs to be parties to the SA MTA *implicitly* changed the law to bestow juristic personhood upon HRECs. We suggest that this counter-argument is flawed – if it was indeed the Minister’s intention to bestow juristic personhood upon HRECs, the action is ultra vires and invalid. Our reasons are as follows: Although the NHA gives the Minister broad powers to regulate matters related to health in South Africa, the capacity to bestow juristic personhood on HRECs is not one of these powers. The SA MTA is delegated legislation and, as such, cannot regulate matters outside of the framework established by the enabling legislation (the NHA). Accordingly, the Minister will be acting ultra vires if he attempts to change the law implicitly to bestow juristic personhood on HRECs through the SA MTA. A more likely possibility, we suggest, is that the requirement in the SA MTA that HRECs must be parties to MTAs is ill-considered and is an oversight on the part of the Minister’s legal advisors.

Labuschaigne et al. argue in support of HRECs being parties to MTAs for two main reasons: first, because HRECs are in a position to fulfil an oversight function with regard to MTAs and, secondly, because, in their view, a HREC can act as a neutral third party to a MTA to ensure negotiation in good faith and unbiased implementation [30]. We suggest that these reasons are unconvincing. As discussed above, HRECs have a mandate to ensure that health research complies with ethical standards; this mandate, first and foremost, includes protecting the interests of research participants. Accordingly, HRECs have an important oversight function in respect of MTAs to ensure that there is consistency between the terms of a MTA and what was promised to the research participant during the informed consent procedure. However, this oversight function, we suggest, is a reason for HRECs to *not* be parties to MTAs and to remain super partes. Being a party to MTAs would lead to a situation in which HRECs must exercise oversight over themselves as parties, which clearly is an untenable situation. Furthermore, the notion that HRECs play the role of a neutral third party between its own research institution and another research institution in order to ensure negotiation in good faith and unbiased implementation is entirely outside the mandate of HRECs. In any event, if some kind of neutral mediator is needed to ensure good faith negotiation and unbiased implementation, the mediator need not be made a party to the MTA. We suggest that HRECs should be kept at a proper distance from the negotiation of the terms of MTAs and should not compromise their objectivity by being involved in negotiations.

Being a party to MTAs therefore not only is a legal impossibility in most instances, but also is incompatible with HRECs’ statutory oversight role. To resolve this issue we suggest that the Minister amends the SA MTA to remove reference to HRECs as parties to MTAs. This amendment will not undermine HRECs’ oversight function – on the contrary it will facilitate it.

Lastly, on the topic of HRECs’ oversight role we raise a practical concern. Although the Department of Health Ethics Guidelines require an HREC to have at least one member that is ‘legally qualified’ ([20] p41), this requirement does not necessarily enable the HREC to fulfil its oversight function over MTAs without the need to obtain external legal advice. The exercise of ensuring consistency between the terms of the MTA and the terms of the consent by the research participants, depending on the intricacy of these terms, can require specialist expertise in contract law. Furthermore, the contractual terms in a MTA often relate to various specialised areas within the law such as intellectual property law and medical law, which typically require input from more than one specialist lawyer. As such, when considering a MTA, HRECs should not hesitate to request a legal opinion from their institutions’ internal legal departments, or, where appropriate, from a law firm.

### Research participant consent

The definition of ‘Project’ in the SA MTA declares it to mean ‘the health research project for which the Materials will be used’ ([13] paragraph 2.15). No indication is given as to how broad or how specifically defined the research project must be. In its definition of ‘Informed Consent’ the SA MTA provides that the research participant must (a) give informed consent for the donation of their Materials to the ‘project’ (presumably ‘Project’ with a capital ‘P’ was intended), but also (b) give informed consent on an ongoing basis to whether and how their Materials will be utilised in the (same) ‘Project’, as approved by the HREC from time to time ([13] paragraph 2.12). It therefore appears that a single ‘Project’ can entail multiple different uses of the Materials that may arise from time to time. As such, a ‘Project’ in this context appears to be more akin to a research *programme* which can comprise any number of distinct research *projects* – with new projects added on an on-going basis. Conversely, under the heading ‘Informed Consent’ the SA MTA refers to a ‘project protocol’ (again, presumably ‘Project’ with a capital ‘P’ was intended), which suggests that a Project is something that is associated with a single protocol – id est a distinct research project ([13] paragraph 10.2). Given this paradoxical use of the term ‘Project’, it is difficult to pin down exactly how broad or specific research participant consent must be in terms of the SA MTA. In the interest of clarity, we suggest that the SA MTA be amended to differentiate between a research *programme* and a distinct research *project* within such a programme.

However, what is clear is the introduction of *dynamic* consent – the idea that consent must be obtained on an on-going basis for new uses of the Materials. This is a departure from the position in the Department of Health Ethics Guidelines, which recommend that consent should be ‘broad enough to allow for future and secondary uses of data’ ([20] p31). Because the SA MTA is the more recent piece of secondary legislation, its dynamic consent provisions will take precedence over the provisions of the Department of Health Ethics Guidelines. However, this will only happen when the SA MTA is triggered into application by the transfer of human biological material. In other words, when health research does not entail the transfer of human biological material between research institutions, the consent provisions of the Department of Health Ethics Guidelines remain applicable.

It should be noted that POPIA’s consent provisions will in turn take precedence over the SA MTA. However, as stated in the background section above, it might be premature at this stage to analyse the SA MTA in the light of POPIA.

Lastly, the issue of consent by children requires attention. According to the SA MTA, only research participants with legal capacity to enter into an agreement ([13] paragraph 2.12). In other words, persons of 18 years and above or emancipated children, can consent to their Materials being transferred by a Provider to a Recipient. There is no provision in the SA MTA that (un-emancipated) children who have *legally* provided their Materials in terms of section 71 of the NHA can consent to their Material being transferred by a Provider to a Recipient. This imposition militates against a need for children to be part of health research and clinical trials in order to develop cures *for children*. We suggest that this represents a clear error in the SA MTA that can have dire consequences for children’s health. The SA MTA should be amended to bring it into alignment with section 71 of the NHA.

### Ownership of material

The SA MTA provides that the research participant ‘remains the owner’ of his or her human biological material and the data that is derived ([13] paragraph 3.3). However, as noted in the background above, extant South African law provides that when research participants donate their human biological material, among other reasons, for research, the institution to which the human biological material is donated acquires exclusive rights in the human biological material ([17] regulation 26). By logical extension, the research institution in the absence of a specific contractual arrangement would enjoy exclusive rights in all derivatives from such human biological material, including data generated from it.

The only way to reconcile the SA MTA with extant law is to interpret the ownership provided for in the SA MTA as *nominal* ownership without any rights in the human biological material or the associated data, that is, ownership in name only. This issue might appear to be a legal technicality, but it is worth remembering that the lack of rights *in* the human biological material or the associated data does not mean that the research participant is without rights *related* to these material and data. For instance, the research participant can have rights vis-à-vis the research institution to which the human biological material was donated and to its successor in title. These rights can include the right to be kept abreast of the research that is conducted on the human biological material, the right to refuse that the human biological material be used for further research beyond the initial research project, the right to request that the human biological material be destroyed, and so forth. The point is that a research participant’s rights related to the research that is conducted on the human biological material he or she donated do not depend on being the owner of the donated human biological material, but can be agreed on separately and in detail.

The SA MTA’s ownership provision might have been intended as a symbolic means to acknowledge the significance that is accorded to human biological material in some South African cultures. Also, this provision might have been intended to strengthen the bargaining position of research participants in the allocation of benefits derived from research. However, these possible aims raise the question whether the legal concept of ownership is an appropriate means to accomplish these aims. Ownership may have symbolic value, but in the broader context of the South African legal landscape, as analysed above, ownership’s value is *only* symbolic (qua *nominal* ownership) and therefore can mislead research participants into thinking that they have greater legal rights than they actually do. For instance, if the research receives any public funding, extant South African law explicitly provides that the research institution that is the recipient of such public funding is entitled to own the intellectual property emanating from such research and that research participants do not by virtue of being research participants qualify for any ownership rights in intellectual property [18].

Apart from potentially being misleading, ownership provisions alone cannot solve the various legal and ethical challenges of a fair distribution of benefits between all the stakeholders in complex transnational research collaboration. More detailed contractual arrangements that carefully are in line with extant law and that clearly specify the rights and duties of parties are required to achieve the aim of a fair distribution of benefits. Accordingly, we suggest that the ownership provision in the SA MTA can do more harm than good and should rather be removed from the SA MTA. As proposed in our discussion of benefit sharing below, best practice guidelines for benefit sharing in South Africa should be developed in consultation with all stakeholders. Such guidelines can provide for other means of symbolic recognition of the significance accorded human biological material in some South African cultures and can provide detailed options to accomplish a fair distribution of benefits that align with extant law.

### Legal fiction of transfer of material

In its current form the SA MTA provides that the HREC must sign the MTA last ([13] paragraph 6.2); and that the MTA becomes effective (only) upon signature by the HREC ([13] paragraph 8). The SA MTA further provides that once the MTA becomes effective, the Material that is the subject of the MTA automatically is deemed to be transferred by the Provider and accepted by the Recipient ([13] paragraph 3.1). These provisions create a legal conundrum: if the Provider *in fact* fails to transfer the Material to the Recipient, in what way would the Recipient enforce its rights in terms of the MTA. Note the Recipient signs that it ‘accepted’ the transfer of the Material. Clearly this situation is an untenable position in which the parties find themselves. We suggest that the SA MTA be amended to remove the deemed-transfer-and-acceptance provision and replace it with a standard provision that enables the parties to determine their own dates of transfer. Also, it would be useful for the parties to stipulate in their MTA exactly how the human biological material and associated data should be de-identified pre-transfer, how the human biological material should be shipped and how the associated data should be made accessible.

### Benefit sharing

The SA MTA requires that benefit sharing between the Provider and Recipient must be ‘discussed and negotiated’ before any Material may be transferred ([13] paragraph 7.1). This requirement is vague: the words ‘discussed and negotiated’ do not mean the same as ‘having reached agreement’. In fact, the words ‘discussed and negotiated’ do not actually require a benefit sharing agreement. We suggest that parties to a MTA should ensure that indeed they have reached agreement regarding benefit sharing before signing the MTA and attach and incorporate their benefit-sharing agreement to the MTA. In the interest of legal certainty we suggest that the SA MTA be amended to replace ‘discussed and negotiated’ with the term ‘agreed’.

As discussed in the background section above, the NHA provides that compensation for giving human biological material for research must be limited to reasonable costs and that to receive *any form of financial or other reward* is a criminal offence ([14] section 60(4),(5)). Accordingly, some possible kinds of benefit sharing clearly are not lawful options. Against this background, the SA MTA could have provided useful guidance regarding the legally-acceptable and ethically-preferable content of substantive terms of a benefit-sharing agreement at the levels of individual research participants, their communities (where relevant), the health system, and local researchers. We suggest that best practice guidelines should be developed in consultation with stakeholders.

### Dispute resolution

The SA MTA provides that in the event that a dispute cannot be resolved amicably any party can institute *action* in accordance with South African law in a South African court, *unless* the parties agree to resolve such dispute by arbitration in terms of a separate arbitration agreement ([13] paragraph 11.3). This provision is problematic in at least two respects: First, in law instituting an *action* has a technical meaning which refers to a specific type of civil procedure. The alternative to an action is an *application* procedure, which should be utilised when no material factual dispute is foreseen and which is considerably quicker and cheaper than an action procedure. However, the SA MTA does not make provision for a quicker and cheaper court procedure, which oversight should be remedied by amending the SA MTA to provide that in the event that a dispute cannot be resolved amicably any party can institute an action *or application*.

The second issue taken with paragraph 11.3 of the SA MTA is that it is unclear whether an arbitration agreement can change the legal system and place of adjudication to a jurisdiction other than South Africa. If the answer is that parties indeed legally can elect to use an arbitration agreement to opt out of South African law and out of adjudication in South Africa, a party that has more negotiating power can use its power to insist on an arbitration agreement that stipulates a legal system, arbitration rules and place of adjudication that benefit it. It is unlikely many South African research institutions have the resources to finance protracted and expensive arbitration in a foreign jurisdiction. From a South African perspective this situation clearly is an undesirable outcome.

Labuschaigne et al. seem to accept that an arbitration agreement indeed can change the legal system and place of adjudication to a jurisdiction other than South Africa [30]. Although Labuschaigne et al. recognise that arbitration abroad may be unaffordable to South African parties, nevertheless they take the position that the arbitration option is necessary because in their view it is ‘in keeping with standards prescribed by Western counterparts’ [30]. This argument raises important policy questions, such as: Should South African policy making be prescribed to by entities in other countries? Is it not the purpose of the SA MTA to protect the interests, inter alia, of the South African research community? On the other hand, it is unreasonable to expect foreign research collaborators, whether they are Providers or Recipients, always to agree to South African jurisdiction over disputes.

We suggest that a fair solution would be that the jurisdiction of the Provider as a general rule should be the applicable legal system and place of adjudication for any disputes related to the MTA – whether through litigation in the courts or through arbitration. The main reason behind the proposal is that it would ensure greater access to justice for research participants in that research participants are likely to be in the same country as the Provider. In the event that research participants have a direct and material interest in a dispute between a Provider and a Recipient they would be able to join the dispute *in their own country* rather than in a foreign country and with a foreign legal system. However, our suggested solution should not be interpreted as a rigid rule; parties should be allowed to approach the Minister (or his delegate) to grant an exemption to the general rule in cases of merit.

### Intellectual property

The navigation of intellectual property law is complex and rife with opposing interests. However, the SA MTA provides simply that intellectual property will be dealt with through ‘relevant laws’ and ‘third-party agreements, as far as there are any’ ([13] paragraph 12). It is important to note that the SA MTA stipulates that the MTA will embody and constitute the ‘entire agreement’ between the parties, which would exclude third-party agreements ([13] paragraph 17.1). Accordingly, all the relevant third-party agreements should be attached as annexures to the MTA and their content explicitly incorporated into the MTA. Absent this measure, a third-party agreement is likely to be held as not binding on the other party to a MTA.

In copyright law, a database is a type of ‘literary work’ and automatically qualifies for copyright protection [32]. Given that data associated with human biological material typically are in the form of a database, such data (as compiled in the database) are the intellectual property of the research institution where the database was created and is independent of any rights regarding the individual bits of data that comprise the database. In addition, data (as compiled in the database) also, depending on the business model of the research institute where the database was created, may qualify as a trade secret of the research institute. Accordingly, in the event that data associated with human biological material is to be transferred, the parties should be acutely aware that they are dealing with already-existing intellectual property. Core considerations, among others, are: Does the Provider assign ownership of the data (as compiled in the database) to the Recipient, give a (non-exclusive or exclusive) right to use (a use-licence) or simply disclose the data on a confidential basis? What are the rights of the Provider in any intellectual property that may be created by the Recipient? How will the research results be commercialised? How will publications be coordinated, and how will authorship be determined?

It is unfortunate that the SA MTA does not give any guidance to the South African research community regarding recommended (or best practice) intellectual property provisions. After all, intellectual property is the currency of the knowledge economy and is something that South Africa should prudently manage. Furthermore, intellectual property and its use also are linked to benefit sharing. If the benefits of health research must be fairly shared, it would be helpful if the SA MTA provided guidance regarding the ways intellectual property and its use should be directed to achieve a fair outcome. We suggest that best practice guidelines for intellectual property provisions be developed in consultation with stakeholders.

### Data privacy

The SA MTA appears to offer broad confidentiality protection ([13] paragraphs 5.2, 13). However, we suggest that the protection is insufficiently specific and overbroad. For instance, the SA MTA provides that the Provider and the Recipient shall treat ‘all information’ relating to the ‘nature and processes of the research’ as confidential ([13] paragraph 13.3). This proposition means that the parties may not reveal even the nature of the research project, such as ‘research on HIV’, to a potential grant funder. We suggest that clearly this provision is overbroad and should be remedied through amendment by the Minister to include the following qualification at the end of the confidentiality provision: ‘ … subject to necessary disclosure of any information in the ordinary course of business, including for purposes of funding applications, complying with funding requirements, and as specified in the Recipient’s research protocol’.

As discussed in the background above, the protection of personal information will be governed by a statute that has been developed specifically for this purpose, POPIA [21]. The SA MTA can complement POPIA and facilitate its implementation. In cases where a Provider in South Africa intends to transfer human biological material or associated data to a Recipient in a foreign country and that foreign country does not provide an ‘adequate level of protection for the processing of personal information’, the Provider will have to obtain prior authorisation for the intended transfer from the Information Regulator ([21] section 57(1)(d)). In such cases a solution would be to contractually bind the foreign Recipient to specific terms that are aligned with POPIA – in effect identify and include all the provisions of POPIA that are relevant to the transfer of human biological material or associated data as contractual terms in a MTA. In this context a revised SA MTA might play an important role; it would greatly assist the South African research community if the relevant POPIA provisions can be condensed to establish contractual terms by privacy law experts, be officially approved by the Information Regulator, and included as standard terms in a revised SA MTA. We suggest that the Minister of Health should consider initiating such a project.

## Conclusions

It seems reasonable to entertain the notion of a national standard MTA that addresses the legal concerns specific to South Africa, that is respectful of local culture and that assists generally South African research institutions and their international collaborators in complying with the law and share the benefits of research in a fair way. However, the manner in which it has been put into practice by the SA MTA is deeply problematic in terms of its substantive fit with the broader legal environment in South Africa, its clarity and its legal practicality. Although some of the issues are complex and require consultative processes with stakeholders and others, to develop comprehensive solutions, most of the issues that we have identified in the SA MTA as problematic can be resolved immediately through relatively simple amendments by the Minister. Table 1 presents a summary of our proposed amendments to improve the SA MTA.

**Table 1:** Summary improve the SA MTA

	Provision	Proposed amendment
**1**	**Application**	
	Notice by the Minister	Replace the words ‘biological material’ with ‘human biological material and associated data’.
**2**	**Health Research Ethics Committees**	
	Recital of parties, paragraph 2.14 and throughout the SA MTA	Remove HRECs as parties.
**3**	**Consent**	
	Paragraph 2.15 and throughout the SA MTA Paragraph 2.12	Differentiate between a research programme and research projects in a programme. Replace the phrase ‘(with legal capacity to do so)’ with ‘and/or the research participant’s parents or guardians in terms of the National Health Act’.
**4**	**Ownership in Material**	
	Paragraph 3.3	Strike out the phrase: ‘and the donor remains the owner of the material until such materials are destroyed.’
**5**	**Transfer of Material**	
	Paragraph 6.2	Replace the current paragraph with the following: ‘The Provider will deliver the Materials to the Recipient according to the following schedule, and in the following media or formats: …’
**6**	**Benefit sharing**	
	Paragraph 7.1	The words ‘discussed and negotiated’ must be replaced with ‘agreed’. (In the longer term) Best practice guidelines to be developed in consultation with stakeholders.
**7**	**Dispute resolution**	
	Paragraph 11.3	Insert a new paragraph 11.2A that reads: ‘In the event that the Provider is located in South Africa, this agreement will be interpreted according to the law of South Africa.’ Insert a new paragraph 11.2B that reads: ‘In the event that the Provider is located in South Africa the division of the high court of South Africa where the Provider is located will have jurisdiction over any dispute related to this agreement except if the parties agree to arbitration, in which case the arbitration hearing must take place within the jurisdiction of the division of the high court of South Africa mentioned above.’ Insert a new paragraph 11.2C that reads: ‘The Parties may only depart from the provisions of paragraph 11.2A and 11.2B above if written permission by the Minister is obtained, in which event the letter of permission must be attached hereto.’ Insert a new paragraph 11.2D that reads: ‘Cognisant of the principles contained in paragraphs 11.2A to 11.2C above, the Parties record their agreement that this Agreement will be interpreted according to the law of … [insert name of jurisdiction], and that any dispute related to this agreement will be adjudicated in … [insert name of level of court and jurisdiction].’ Replace the phrase in paragraph 11.3 ‘either Party may institute action in accordance with South African laws, in a South African court, unless the Parties agree to resolve such dispute by arbitration in terms of a separate arbitration agreement’ with ‘either Party may institute legal proceedings in a court that has jurisdiction, unless the Parties agree to resolve such dispute by arbitration in terms of a separate arbitration agreement, subject to the provisions of paragraphs 11.2A to 11.2D above.’
**8**	**Intellectual property**	
	Paragraph 12	Insert a new paragraph 12A that all relevant third-party agreements must be listed in the MTA, attached to the MTA, and that their content is deemed to be incorporated into the MTA. (In the longer term) Best practice guidelines to be developed in consultation with stakeholders.
**9**	**Data Privacy**	
	Paragraph 13.3	Replace the current paragraph with the following: ‘The Provider and the Recipient shall treat all information relating to the nature and processes of the research in whatever form as confidential, subject to necessary disclosure of any information in the ordinary course of business, including for purposes of funding applications, complying with funding requirements, and as specified in the Recipient’s research protocol.’ (In the longer term) The relevant POPIA provisions should be condensed to contractual terms by privacy law experts, officially approved by the Information Regulator and included as standard terms in a revised SA MTA.

## Data Availability

Not applicable.

## Abbreviations

Department of Health Ethics Guidelines: Department of Health of South Africa. *Ethics in Health Research: Principles, Processes and Structures*. 2nd ed. Pretoria: Department of Health, 2015
DTA: Data transfer agreement
HIV: Human immunodeficiency virus
HREC: Health research ethics committee
Minister: South African Minister of Health
MTA: Material transfer agreement
NHA: Government of South Africa. National Health Act 61 of 2003
NIH: National Institutes of Health
OECD: Organisation for economic cooperation and development
POPIA: Government of South Africa. Protection of Personal Information Act 4 of 2013
Provider: A party to a material transfer agreement that intends to provide human biological material to the other party, the recipient
Recipient: A party to a material transfer agreement that intends to receive the material provided by the other party, the provider, and to use it for research purposes
SA: South Africa or South African
SA MTA: Government of South Africa. Material Transfer Agreement for Human Biological Materials Government Notice 719, Government Gazette 41,781 of 20 July 2018
SMTA 2: Standard material transfer agreement 2
TTO: Technology transfer office
UB MTA: Uniform biological material transfer agreement
US: United States
WHO: World Health Organisation
