# Correction to: South Africa’s new standard material transfer agreement: proposals for improvement and pointers for implementation

**DOI:** 10.1186/s12910-020-00547-6

**Published:** 2020-11-20

**Authors:** Donrich W. Thaldar, Marietjie Botes, Annelize Nienaber

**Affiliations:** 1 School of Law, University of KwaZulu-Natal, Durban, South Africa; 2 African Health Research Flagship, University of KwaZulu-Natal, Durban, South Africa; 3 Faculty of Law, University of Pretoria, Pretoria, South Africa; 4 Centre for Ethics and Philosophy of Health Sciences, Faculty of Health Sciences, University of Pretoria, Pretoria, South Africa; 5 Division of Law, Abertay University, Dundee, Scotland, UK

## Correction to: BMC Medical Ethics (2020) 21:85 https://doi.org/10.1186/s12910-020-00526-x

It was highlighted that the original article contained an alignment typesetting error in Table 1. This Correction article shows the correct Table 1. The original article has been updated.

**Table 1:** Summary of proposed amendments to improve the SA MTA

	Provision	Proposed amendment
1	**Application**	
	Notice by the Minister	Replace the words ‘biological material’ with ‘human biological material and associated data’.
2	**Health Research Ethics Committees**	
	Recital of parties, paragraph 2.14 and throughout the SA MTA	Remove HRECs as parties.
3	**Consent**	
	Paragraph 2.15 and throughout the SA MTA	Differentiate between a research programme and research projects in a programme.
	Paragraph 2.12	Replace the phrase ‘(with legal capacity to do so)’ with ‘and/or the research participant’s parents or guardians in terms of the National Health Act’.
4	**Ownership in Material**	
	Paragraph 3.3	Strike out the phrase: ‘and the donor remains the owner of the material until such materials are destroyed.’
5	**Transfer of Material**	
	Paragraph 6.2	Replace the current paragraph with the following: ‘The Provider will deliver the Materials to the Recipient according to the following schedule, and in the following media or formats:…’
6	**Benefit sharing**	
	Paragraph 7.1	The words ‘discussed and negotiated’ must be replaced with ‘agreed’. (In the longer term) Best practice guidelines to be developed in consultation with stakeholders.
7	**Dispute resolution**	
	Paragraph 11.3	Insert a new paragraph 11.2A that reads: ‘In the event that the Provider is located in South Africa, this agreement will be interpreted according to the law of South Africa.’ Insert a new paragraph 11.2B that reads: ‘In the event that the Provider is located in South Africa the division of the high court of South Africa where the Provider is located will have jurisdiction over any dispute related to this agreement except if the parties agree to arbitration, in which case the arbitration hearing must take place within the jurisdiction of the division of the high court of South Africa mentioned above.’ Insert a new paragraph 11.2C that reads: ‘The Parties may only depart from the provisions of paragraph 11.2A and 11.2B above if written permission by the Minister is obtained, in which event the letter of permission must be attached hereto.’ Insert a new paragraph 11.2D that reads: ‘Cognisant of the principles contained in paragraphs 11.2A to 11.2C above, the Parties record their agreement that this Agreement will be interpreted according to the law of … [insert name of jurisdiction], and that any dispute related to this agreement will be adjudicated in … [insert name of level of court and jurisdiction].’ Replace the phrase in paragraph 11.3 ‘either Party may institute action in accordance with South African laws, in a South African court, unless the Parties agree to resolve such dispute by arbitration in terms of a separate arbitration agreement’ with ‘either Party may institute legal proceedings in a court that has jurisdiction, unless the Parties agree to resolve such dispute by arbitration in terms of a separate arbitration agreement, subject to the provisions of paragraphs 11.2A to 11.2D above.’
8	**Intellectual property**	
	Paragraph 12	Insert a new paragraph 12A that all relevant third-party agreements must be listed in the MTA, attached to the MTA, and that their content is deemed to be incorporated into the MTA. (In the longer term) Best practice guidelines to be developed in consultation with stakeholders.
9	**Data Privacy**	
	Paragraph 13.3	Replace the current paragraph with the following: ‘The Provider and the Recipient shall treat all information relating to the nature and processes of the research in whatever form as confidential, subject to necessary disclosure of any information in the ordinary course of business, including for purposes of funding applications, complying with funding requirements, and as specified in the Recipient’s research protocol.’ (In the longer term) The relevant POPIA provisions should be condensed to contractual terms by privacy law experts, officially approved by the Information Regulator and included as standard terms in a revised SA MTA.

## References

[1] Thaldar (2020). BMC Medical Ethics.

